# SARS-CoV-2 recombinant proteins stimulate distinct cellular and humoral immune response profiles in samples from COVID-19 convalescent patients

**DOI:** 10.6061/clinics/2021/e3548

**Published:** 2021-11-23

**Authors:** Laís Teodoro da Silva, Marina Mazzilli Ortega, Bruna Tiaki Tiyo, Isabelle Freire Tabosa Viana, Tayná Evily de Lima, Tania Regina Tozetto-Mendoza, Luanda Mara da Silva Oliveira, Franciane Mouradian Emidio Teixeira, Roberto Dias Lins, Alexandre de Almeida, Maria Cassia Mendes-Correa, Alberto Jose da Silva Duarte, Telma Miyuki Oshiro

**Affiliations:** I Laboratorio de Investigacao Medica em Dermatologia e Imunodeficiencias (LIM 56), Hospital das Clinicas HCFMUSP, Faculdade de Medicina, Universidade de Sao Paulo, Sao Paulo, SP, BR.; II Laboratorio de Virologia (LIM-52), Hospital das Clinicas HCFMUSP, Faculdade de Medicina, Universidade de Sao Paulo, Sao Paulo, SP, BR.; III Departamento de Virologia, Instituto Aggeu Magalhaes, Fundacao Oswaldo Cruz, Recife, PE, BR.; IV Divisao de Laboratorio Central, Hospital das Clinicas HCFMUSP, Faculdade de Medicina, Universidade de Sao Paulo, Sao Paulo, SP, BR.

**Keywords:** SARS-CoV-2, COVID-19, Antigens, Immune Response

## Abstract

**OBJECTIVES:**

In this preliminary study we investigated cellular and humoral immune responses to severe acute respiratory syndrome coronavirus 2 (SARS-CoV-2) antigens in blood samples from 14 recovered coronavirus disease 2019 (COVID-19) patients and compared them to those in samples from 12 uninfected/unvaccinated volunteers.

**METHODS:**

Cellular immunity was assessed by intracellular detection of IFN-γ in CD3+ T lymphocytes after stimulation with SARS-CoV-2 spike (S1), nucleocapsid (NC), or receptor-binding domain (RBD) recombinant proteins or overlapping peptide pools covering the sequence of SARS-CoV-2 spike, membrane and nucleocapsid regions. The humoral response was examined by ELISAs and/or chemiluminescence assays for the presence of serum IgG antibodies directed to SARS-CoV-2 proteins.

**RESULTS:**

We observed differences between humoral and cellular immune profiles in response to stimulation with the same proteins. Assays of IgG antibodies directed to SARS-CoV-2 NC, RBD and S1/S2 recombinant proteins were able to differentiate convalescent from uninfected/unvaccinated groups. Cellular immune responses to SARS-CoV-2 protein stimuli did not exhibit a specific response, as T cells from both individuals with no history of contact with SARS-CoV-2 and from recovered donors were able to produce IFN-γ.

**CONCLUSIONS:**

Determination of the cellular immune response to stimulation with a pool of SARS-CoV-2 peptides but not with SARS-CoV-2 proteins is able to distinguish convalescent individuals from unexposed individuals. Regarding the humoral immune response, the screening for serum IgG antibodies directed to SARS-CoV-2 proteins has been shown to be specific for the response of recovered individuals.

## INTRODUCTION

Coronavirus disease 2019 (COVID-19) emerged as a pandemic in March 2020 once severe acute respiratory syndrome coronavirus 2 (SARS-CoV-2) became widespread worldwide. SARS-CoV-2 is a member of the Coronaviridae family and betacoronavirus subfamily, as along with the already known highly pathogenic viruses severe acute respiratory syndrome coronavirus (SARS-CoV) and Middle East respiratory syndrome coronavirus (MERS-CoV). These viruses are enveloped and composed of a positive-sense single-stranded RNA (+ssRNA) genome (1) and phosphorylated nucleocapsid protein. The spike (S), membrane (M) and envelope (E) proteins are located on the phospholipid bilayer membrane surrounding the viral particle (2).

Knowledge of the immune status of a population is essential in estimating the number of people who have had contact with the virus and evaluating the effectiveness of vaccines. Additionally, the emergence of new SARS-CoV-2 variants indicates the need to continuously monitor the population's immune status. Moreover, even after the beginning of massive vaccination in some countries, aspects related to the definition of correlates of protection against SARS-CoV-2 infection remain unclear. In this context, it is crucial to understand how the immune system acts and controls the infection and which immune mechanisms could be explored to prevent reinfections.

In severe COVID-19, the immune system displays an imbalance characterized by lymphopenia and an activated lymphocyte profile or dysfunction. Changes in T cell phenotypes are also observed and can include not only the expression of cell markers, such as CD38, HLA-DR and CD40L, but also production of cytokines, such as IL-6, TNF-α and IFN-γ (3-6). A reduced number of CD4+ and CD8+ T cells is often associated with exhausted T cells exhibiting low proliferative ability and increased levels of proinflammatory cytokine production (7). In the acute phase, SARS-CoV-2-specific T cells exhibit an activated cytotoxic phenotype, while in convalescent patients, these cells show a memory phenotype (8).

T cells perform multiple functions associated with viral control, including cytotoxic activity and the production of immune mediators to enable control or virus clearance (9,10). In this context, the Th1 cytokine IFN-γ can inhibit viral replication by inducing cytotoxic function of CD8+ T cells and promoting the production of other cytokines, such as TNF-α and IL-2 (10).

In addition, B cells also play an important role in producing transient IgM and persistent IgG responses against the virus (11). Antibody responses to acute viral infection are induced in patients with COVID-19, and the seroconversion rate rapidly increases during the first two weeks of disease (12). Although anti-SARS-CoV-2 IgG levels decrease after 3 months of infection, this fact correlates with disease severity (13).

Several studies have used SARS-CoV-2 antigens (3,14-19) to stimulate a T cell response in *in vitro* assays. Although it has been demonstrated that T cell responses are focused not only on the S protein but also on the SARS-CoV-2 M and N regions (15), no previous studies have compared T cell response specificity between SARS-CoV-2 peptide pools (M, N and S peptides together) and proteins.

Here, we investigated cellular and humoral specific immune responses to SARS-CoV-2 antigens in COVID-19 convalescent subjects compared to those in uninfected/unvaccinated controls. Concerning the T cell immune response, differences were observed in the studied groups regarding the specificity of IFN-γ production after stimulation with SARS-CoV-2 peptides or with proteins. Furthermore, distinct profiles were observed between the cellular and humoral responses to SARS-CoV-2 proteins in convalescent individuals.

## MATERIALS AND METHODS

### Study subjects

We analyzed 14 convalescent COVID-19 patients (as confirmed by a positive RT-PCR test) and 12 noninfected/unvaccinated donors (confirmed by a negative serology via anti-SARS-CoV-2 IgG test (LIAISON^®^ SARS-CoV-2 S1/S2 IgG, DiaSorin S.p.A., VC, IT) at the time of inclusion in the study). The characteristics of recruited individuals are described in Table 1. Written informed consent was obtained according to protocols of the Clinics Hospital Ethical Committee (CAPPesq) (São Paulo, Brazil) under the approval protocol number 4.360.357. All participants gave informed consent at the time of recruitment into the study.

### Antigens

#### SARS-CoV-2 proteins

Commercial SARS-CoV-2 spike (S1) (Z03501) and nucleocapsid (NC) (Z03488) proteins (both purchased from GenScript (NJ, USA)) and the recombinant receptor-binding domain (RBD) of the SARS-CoV-2 spike protein (Wuhan strain) (kindly provided by Instituto Aggeu Magalhães - Fiocruz) were used to study humoral and cellular responses.

#### SARS-CoV-2 peptide pools

Overlapping peptide pools (OPPs) (15-mers with 11 amino acid overlap) covering the immunodominant sequence domain representing spike (S) (PepTivator^®^ SARS-CoV-2 Prot_S; #130-126-700) and the complete sequence of the membrane (M) (PepTivator^®^ SARS-CoV-2 Prot_M; #130-126-702) and nucleocapsid (NC) (PepTivator^®^ SARS-CoV-2 Prot_N; #130-126-698) SARS-CoV-2 proteins (Miltenyi Biotec, CA, USA) were used to study cellular responses.

### T cell responses to SARS-CoV-2 antigens

To study the T cell response against SARS-CoV-2 antigens, 1x10^6^ freshly collected PBMCs/well were maintained at 37°C in an atmosphere containing 5% CO_2_ in RPMI-1640 with 10% fetal bovine serum (Gibco; Thermo Fisher Scientific, OR, USA). Cells were stimulated with the recombinant protein S1 (50 ng/mL) or NC (60 ng/mL) or a mixture of both SARS-CoV-2 proteins (25 ng/mL each), as well as recombinant RBD of the SARS-CoV-2 protein (500 ng/mL). In some assays, cells were stimulated separately with OPPs at a concentration of 1 ug/mL or assayed using 0.3 µg/mL each OPP (S, M and N), totaling a final concentration of 1 μg/mL. These protein and peptide concentrations were defined in previous assays (data not shown). The positive control was composed of lymphocytes stimulated with phorbol myristate acetate (30 ng/mL, PMA; Sigma-Aldrich) and ionomycin (0.3 μg/mL; Sigma-Aldrich) for the last 6h, while the negative control comprised only PBMCs (without stimulus). The percentage of cytokine-producing lymphocytes was subtracted from the negative control group. A percentage of IFN-γ-producing T cell responses below 0.001% was considered negative (20).

**Figure S1:**
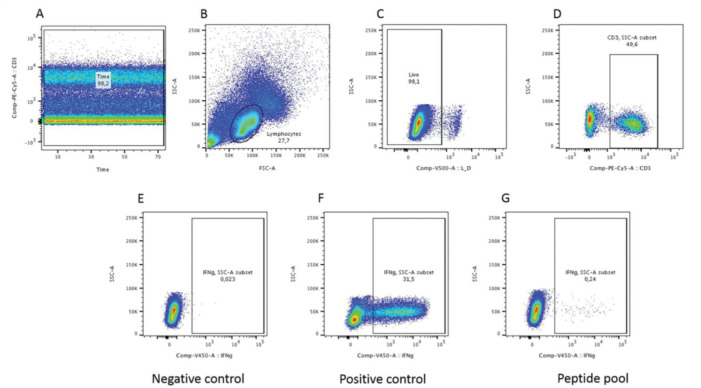
Representative flow cytometry plots of IFN-γ expression by CD3^+^ T cells from one convalescent COVID-19 individual. (A) The PE-Cy5 x time analysis strategy was employed to exclude possible unstable cell flow, after which size and granularity patterns of cells were analyzed by FSC and SSC (B) and therefore within the live cell population (C). CD3^+^ T cells were analyzed (D) for IFN-γ production in the negative control (without stimulation) (E) or following stimulation with phorbol myristate acetate and ionomycin (positive control) (F) or SARS-CoV-2 peptide pools (G). Samples were acquired on an LSR Fortessa (BD). Flow cytometry data were analyzed using FlowJo v.10 software.

The cells were stained with an amine-reactive fixable live*/*dead stain (Gibco; Life Technologies), anti-CD3 PE-Cy5 antibody (clone 7D6, Invitrogen; Thermo Fisher Scientific) and intracellular marker IFN-γ V450 (clone B27, BD Biosciences, CA, USA) using a Cytofix/Cytoperm kit (BD Bioscience) as recommended by the manufacturer. An average of 200.000 T lymphocytes was acquired on an LSR Fortessa (BD Bioscience), and the analysis was performed using FlowJo v. 10.6.1 software (Ashland, OR: Becton, Dickinson and Company) (Figure S1).

### Detection of anti-S1/S2 IgG antibodies through chemiluminescence assay

An automated quantitative chemiluminescence assay was performed for determination of IgG antibodies to the S1 and S2 proteins of SARS-CoV-2 using LIAISON^®^ tests (DiaSorin S.p.A., Saluggia, Italy). This assay was validated with samples from 162 patients and had a sensitivity of 97.8% and specificity of 99.1% after 14 days of symptoms.

### Detection of anti-RBD IgG antibodies through ELISA

High binding, half area 96-well polystyrene plates (Costar; Lowell, MA, USA) were coated overnight at 4°C with 1 μg/mL recombinant SARS-CoV-2 RBD protein diluted in 0.2 M carbonate/bicarbonate buffer (Pierce, IL, USA). The RBD was produced in eukaryotic cells according to a protocol described elsewhere (21) and purified through affinity chromatography. The plates were blocked with skim milk (Bio-Rad) at 5% (w/v) in PBS-T buffer [1X PBS with 0.05% (v/v) Tween 20] for 1h at room temperature. Serum samples were diluted (1:50) in assay buffer [5% (w/v) skim milk in PBS-T] and added to the plates. All samples were incubated for 2h at room temperature. The plates were washed five times with PBS-T, followed by incubation with horseradish peroxidase (HRP)-linked antibody against total IgG (Jackson ImmunoResearch, 1:30,000 dilution) diluted in assay buffer for 1h at room temperature. After a second wash step, the reaction was developed by the addition of tetramethylbenzidine TMB-KPL substrate (Pierce, IL, USA) for 30 min at room temperature, followed by 1 N HCl. Optical densities at a wavelength of 450 nm (OD_450 nm_) were read using a microplate spectrophotometer (BioTek; Winooski, VT, USA). A subset of COVID-19 positive (as determined by RT-PCR and serology) and SARS-CoV-2-naïve sera were used as positive and negative controls, respectively. Positive and negative controls were run in each plate and used to determine the reproducibility of the assay. Accuracy of the ELISA was determined by testing a set of 100 COVID-19-negative and 53 COVID-19-positive serum samples. Serum samples were considered positive for anti-RBD IgG antibodies when the sample absorbance/negative control ratio ≥4.139, corresponding to a sensitivity of 92.45% and specificity of 97%. ELISA data were analyzed by GraphPad Prism v.7 software (GraphPad Software, San Diego CA, USA).

### Detection of anti-NC IgG antibodies through ELISA

Anti-SARS-CoV-2 NC antibodies were detected by an ELISA protocol whose assay sensitivity was 90.3% and specificity was 97.9%, as described by Tozetto-Mendoza et al. (22). Briefly, 96-well microplates were coated with a 46-kDa protein derived from recombinant SARS-CoV-2 NC protein (GenScript Inc., NJ, USA) at a concentration of 100 ng/mL. The serum samples were used at a dilution of 1:200 and added for 1h at room temperature. After rinsing cycles, the wells were incubated with horseradish peroxidase-conjugated anti-human IgG (Sigma Aldrich, San Louis, MO, USA) diluted to an optimal titred of 1:20,000 in skim milk (Bio-Rad) at 5% (w/v) in PBS-T buffer. After rinsing, the chromogenic substrate TMB (Siemens Healthcare, Marburg, Germany) was added, and the plates were incubated at room temperature for 15 min. The reaction was stopped by the addition of 2 N H_2_SO_4_ and the optical density in individual wells was measured at 450/650 nm in an automatic ELISA reader (Biochrom Ltd., Asys Expert Plus Microplate Reader, Cambridge, UK). The cut-off value was designated as the mean optical density and three standard deviations of 94 control serum samples. Data were expressed as the reactivity index (RI), which was calculated by dividing sample optical density by the cut-off value. Samples were considered positive when the RI value ≥1.

### Statistical analysis

To compare the two studied groups, Student’s t-test with a nonparametric Mann-Whitney test was performed using GraphPad Prism v.8 software (GraphPad Software). *p*<0.05 was considered statistically significant.

## RESULTS

### Characteristics of volunteers

We included 14 convalescent COVID-19 individuals of both sexes who had been diagnosed as positive for COVID-19 according to RT-qPCR assay of a nasal/oral swab sample at least 30 days before the inclusion in the study and confirmed by serological detection of anti-SARS-CoV-2 IgG antibody. Two convalescent individuals (CONV_6 and CONV_11) were not tested by RT-qPCR assay during COVID-19 symptoms, and they were confirmed only by positive anti-SARS-CoV-2 IgG test; one of them (CONV_11) presented reduced antibody levels at the time of inclusion in the study. Regarding the composition of the negative control group, 12 individuals with no history of COVID-19 or vaccination for COVID-19 were included, which was confirmed by negative serology for SARS-CoV-2 at the time of inclusion in the study. The data of study volunteers are presented in Table 1.

The age of volunteers varied considerably (**p<0.01), between 22 and 75 years old. We did not find any correlation between the age of the recruited volunteers and the analyzed parameters, such as, disease severity, antibody titers and IFN-γ production (data not shown). The time elapsed between infection and collection to assess the cellular response ranged from 1 to 10 months, and the clinical form also varied among asymptomatic, mild, and moderate symptoms or more severe forms, requiring hospitalization.

### IFN-γ production by T lymphocytes stimulated with SARS-CoV-2 recombinant proteins

**Figure 1:**
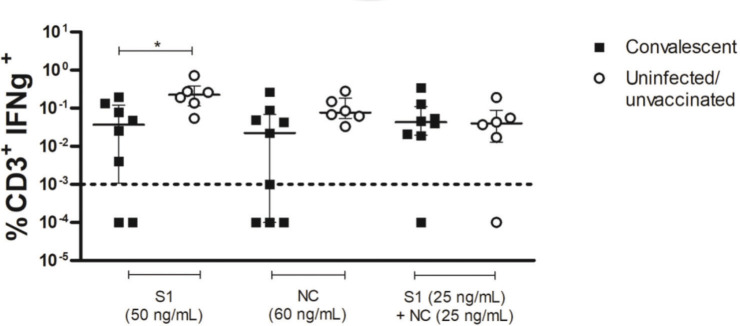
T cell responses to SARS-CoV-2 proteins. PBMCs from convalescent COVID-19 individuals (black squares) (n=9) and from uninfected/unvaccinated donors (white circles) (n=6) were stimulated for five days with 50 ng/mL SARS-CoV-2 spike (S1); 60 ng/mL nucleocapsid (NC); or a mixture of both SARS-CoV-2 proteins (25 ng/mL each). The logarithmic scale represents the percentage of T cells producing IFN-γ. Scatterplots show lines at the median with interquartile ranges. The dashed line represents the assay cut-off value of 0.001%. IFN-γ expression by CD3+ T cells was analyzed by intracellular flow cytometry. T-tests and nonparametric Mann-Whitney tests were used to calculate *p* values. Asterisks denote statistically significant differences between the groups (*p<0.05).

The cellular response in COVID-19 convalescent individuals was determined through detection of intracellular IFN-γ in T lymphocytes stimulated with recombinant spike (S1) and nucleocapsid (NC) proteins individually or pooled (Figure 1). The control group comprised individuals with no previous history of infection or vaccination against COVID-19.

**Figure S2:**
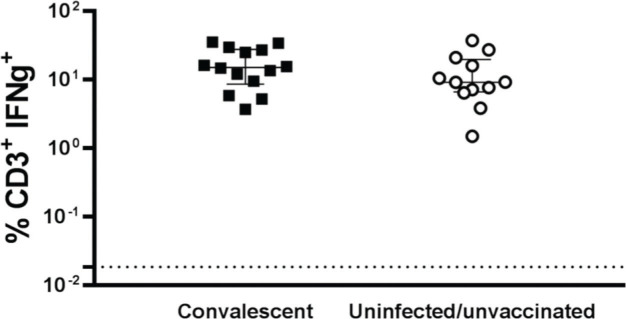
T cell responses to PMA/ionomycin. PBMCs from convalescent COVID-19 individuals (black squares) (n=14) and uninfected/unvaccinated donors (white circles) (n=12) were incubated for the last 6h with PMA (30 ng/mL) and ionomycin (0.3 μg/mL) (positive control). The logarithmic scale represents the percentage of T cells producing IFN-γ. Scatterplots show lines at the median with interquartile ranges. The dashed line represents the assay cut-off value of 0.001%. IFN-γ expression by CD3+ T cells was analyzed by intracellular flow cytometry. T-tests and nonparametric Mann-Whitney tests were used to calculate p values. No significant differences in IFN-γ production were observed between groups (p=0.27).

We observed that stimulation with S1 protein was able to promote the production of IFN-γ in both samples from convalescent individuals and negative controls. Unexpectedly, negative controls showed a significantly higher response than convalescents (*p<0.05). The response to NC stimulus or the protein pool (S1 + NC) was similar between the two groups tested. PBMCs from all donors were able to respond to the positive control (PMA-ionomycin) (Figure S2).

Together, the results suggest that the use of these recombinant proteins to stimulate cells with the aim of evaluating the lymphocyte response to SARS-CoV-2 does not allow differentiation of the response between convalescent and uninfected/unvaccinated individuals.

### IFN-γ production by T lymphocytes stimulated with SARS-CoV-2 peptides

**Figure 2:**
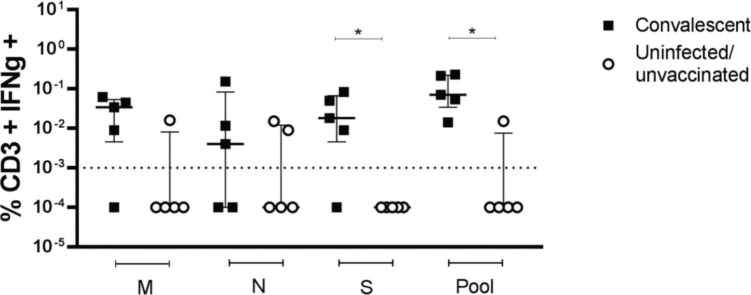
T cell responses to SARS-CoV-2 peptide pools. PBMCs from convalescent COVID-19 individuals (black squares) (n=5) and from uninfected/unvaccinated donors (white circles) (n=5) were incubated for 18h with membrane (M) (1 μg/mL), nucleocapsid (N) (1 μg/mL) or spike (S) (1 μg/mL) peptides individually or a mixture of SARS-CoV-2 protein peptide pools grouped (Pool) at a final concentration of 1 μg/mL. The logarithmic scale represents the percentage of T cells producing IFN-γ. Scatterplots show lines at the median with interquartile ranges. The dashed line represents the assay cut-off value of 0.001%. IFN-γ expression by CD3+ T cells was analyzed by intracellular flow cytometry. T-tests and nonparametric Mann-Whitney tests were used to calculate *p* values. Asterisks denote statistically significant differences between the groups (*p<0.05).

Next, we evaluated the production of IFN-γ by T lymphocytes stimulated with OPPs from viral components, membrane (M), nucleocapsid (NC) and viral spike (S), separately or pooled. The result is shown in Figure 2.

We observed that both the S peptide alone and the pool of 3 peptides were able to discriminate the production of IFN-γ between convalescent individuals and uninfected/unvaccinated controls (*p<0.05). In addition, the use of equivalent amounts of these combined peptides was able to intensify the response in all convalescent individuals.

### Comparison between IFN-γ production by T lymphocytes stimulated with recombinant RBD protein or peptide pool

**Figure 3:**
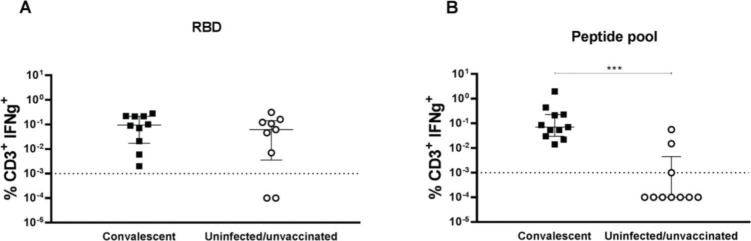
Comparison of IFN-γ production by T cells stimulated with a SARS-CoV-2 recombinant protein or peptide pool. PBMCs from COVID-19 convalescent individuals (black squares) (n=11) and from uninfected/unvaccinated donors (white circles) (n=10) were stimulated for five days with 500 ng/mL RBD protein (A) or were incubated for 18h with a mixture of grouped SARS-CoV-2 peptide pools (M+N+S) at a final concentration of 1 μg/mL (B). The logarithmic scale represents the percentage of T cells producing IFN-γ. Scatterplots show lines at the median with interquartile ranges. The dashed line represents the assay cut-off value of 0.001%. IFN-γ expression by CD3+ T cells was analyzed by intracellular flow cytometry. T-tests and nonparametric Mann-Whitney tests were used to calculate *p* values. Statistically significant differences were observed between the groups when cells were stimulated with SARS-CoV-2 peptide pools (***p<0.001).

Considering the differences in the T cell response of cell cultures in the presence of either SARS-CoV-2 proteins or peptide pools, we compared the response profile of paired cultures (from convalescent individuals or uninfected/unvaccinated controls) stimulated with pooled peptides or recombinant RBD protein (Figure 3).

Our data showed that cell cultures from COVID-19 convalescent individuals exhibited a very heterogeneous response profile in the presence of RBD protein. Notably, the majority of tested individuals were able to produce IFN-γ in response to RBD stimulation. However, an overall similar profile was observed among samples from negative control individuals, with some of them able to respond with the same intensity as the convalescent group.

Convalescent samples stimulated with the peptide pool were able to produce IFN-γ at different levels. Interestingly, the majority of uninfected/unvaccinated individuals had baseline levels of IFN-γ production, confirming that pooled OPPs are more capable than the recombinant proteins tested of stimulating a specific response to SARS-CoV-2 antigens (***p< 0.001).

### Profile of the IgG antibody response directed to SARS-CoV-2 recombinant proteins

**Figure 4:**
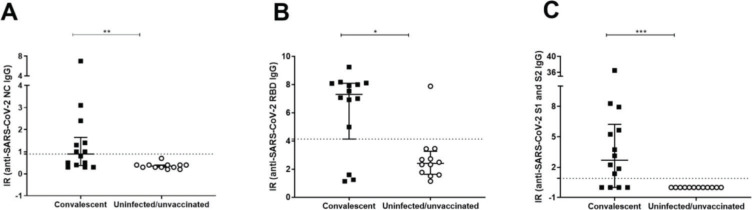
Detection of anti-SARS-CoV-2 specific IgG. Serum samples obtained from COVID-19 convalescent individuals (n=14) and uninfected/unvaccinated donors (n=12) were analyzed to determine the IgG antibody reactivity index (RI) to nucleocapsid (NC) (A), the receptor-binding domain (RBD) (B), and spike (S1 and S2) (C). Scatterplots show lines at the median with interquartile ranges. Dotted lines represent the sample classification cut-off for each test. T-tests and nonparametric Mann-Whitney tests were used to compare the studied groups (*p<0.05; **p<0.005; ***p<0.001).

Once we observed that the cellular response to SARS-CoV-2 recombinant protein is not able to differentiate responses from convalescent and uninfected/unvaccinated individuals, we assessed the profile of IgG antibodies directed to the SARS-CoV-2 nucleocapsid (NC), RBD and S1/S2 proteins in serum samples from the same individuals previously tested for cellular responses (Figure 4).

Our data show that all three assays (detection of anti-NC, anti-RBD and anti-S1/S2 antibodies) were able to differentiate between convalescent and uninfected/unvaccinated groups. Notably, not all convalescent samples were positive for the presence of anti-SARS-CoV-2 antibodies. The data show that 50%, 82.35% and 76.47% of convalescent samples exhibited IgG antibodies against the NC, RBD and S1/S2 proteins, respectively. Notably, all convalescent individuals had a positive diagnosis for COVID-19 confirmed by RT-qPCR over the duration of symptoms or by anti-S1/S2 IgG serology detected one-to-six months after symptom onset. Interestingly, one negative donor (UI / UV_29) showed humoral and cellular responses against the RBD protein, despite having no previous history of infection or vaccination against COVID-19 (Figure 4 and Table 2), which could be due to cross-reaction with the RBD protein of SARS-CoV.

### Comparison between humoral and cellular responses to SARS-CoV-2 antigens

Once we observed differences between humoral and cellular immune profiles in response to stimulation with the same proteins, we decided to compare the obtained results individually, including the analysis of stimulation with peptides, to integrate such aspects.

Regarding the humoral profile, assays using RBD protein were almost in full agreement with reference values, i.e., a SARS-CoV-2 S1/S2 IgG commercial test, except for one sample that tested positive with the RBD but negative by the S1/S2 test. On the other hand, results for anti-NC IgG antibodies were negative in 7 of 14 convalescent samples. Of these samples, 3 were in disagreement with the reference test. All samples of negative controls were in agreement with the reference results (Table 2).

Regarding cell analysis, despite not having completed all the tests for each antigen, which can hinder the interpretation of results, we observed that stimulation with peptides induced a more specific response than stimulation with SARS-CoV-2 proteins. At least in part, these findings were in agreement with the humoral anti-S1/S2 response, with no response in most uninfected/unvaccinated individuals. IFN-γ production was also present in some convalescent samples that tested negative for the presence of antibodies, which suggests distinct profiles between the humoral and cellular responses. Among uninfected/unvaccinated controls, we observed that at least two of them were able to produce IFN-γ in response to stimulation with peptides. However, as expected, other samples tested did not respond to stimulation.

In relation to stimulation with the recombinant proteins S1, RBD or NC, we observed that almost all samples tested, both those from the convalescent and negative control groups, presented IFN-γ production at different levels in response to stimulation with at least one protein tested.

## DISCUSSION

Investigating the anti-SARS-CoV-2 immune response is an important approach to understanding the immune status in a population, estimating the number of people who have already been infected by the virus and evaluating the effectiveness of vaccines.

In this study, blood samples collected from individuals recovered from COVID-19 and uninfected/unvaccinated donors were analyzed for cellular and humoral immune responses against recombinant proteins and/or peptides of SARS-CoV-2. We observed differences in IFN-γ production by T cells after stimulation with peptides and proteins of SARS-CoV-2. Additionally, a distinct profile between cellular and humoral responses to SARS-CoV-2 proteins was noticed.

In a preliminary assay, to analyze the specific cellular response of recovered COVID-19 individuals, we tested the ability of a panel of SARS-CoV-2 recombinant proteins, individually or pooled, to stimulate T cells and evaluated IFN-γ production. We observed that the control group, composed of individuals without a history of infection or vaccination for COVID-19, presented similar and sometimes higher IFN-γ production after stimulation with recombinant proteins than recovered individuals, suggesting that these antigens provide a low specificity profile for the IFN-γ response. The number of individuals tested in this preliminary assay is small, so these results should be evaluated with caution. Next, we tested T cell stimulation using SARS-CoV-2 peptide pools that cover immunodominant epitopes from the structural proteins: spike, membrane and nucleocapsid. For this assay, we observed a more specific response, with a distinct profile between convalescent individuals and the controls. Testing a larger number of individuals with paired samples, we compared the production of IFN-γ between convalescents and controls using recombinant RBD protein and peptides, confirming that the response to peptides was significantly more specific.

Notably, the type of antigen used to assess the cellular or humoral immune response is of utmost importance to determine assay specificity. *In vivo* activation of a T cell population relies on the presentation of the antigen after its processing into small peptides and subsequent loading onto major histocompatibility complex (MHC) class I and II molecules on the surface of antigen-presenting cells (23). Similarly, *in vitro* activation of this population requires T cell receptor engagement by short peptides in solution to mimic natural activation (24). Locking the peptide onto MHC molecules is crucial for receptor recognition since this interaction ensures that the peptide is presented in a stable conformation to allow binding. Accordingly, our data have shown that T cell stimulation with SARS-CoV-2 peptides triggers a more specific response than stimulation with full-length viral proteins, which might indicate that large proteins compromise antigen recognition by T cells and therefore lead to suboptimal responses.

Although we observed a nonspecific response after T cell stimulation with the full-length SARS-CoV-2 NC, RBD and S1 proteins, our data showed that the IgG antibody profiles against these proteins were able to differentiate the convalescent and uninfected groups. This finding indicates that a specific humoral response is induced after COVID-19 infection and is supported by previous studies showing that antibody responses are often directed to conformational epitopes, which are presented only when the target protein is in its native conformation and properly folded (25).

Interestingly, not all recovered patients presented SARS-CoV-2-specific antibodies despite having positive RT-qPCR or positive IgG serology results after symptom onset. Regarding the four convalescent individuals who did not present anti-S1/S2 IgG antibodies at study inclusion, all of them had mild symptoms, two had just reached one month after symptom onset when they were included in the study, and the last two had a positive RT-qPCR or serology result for at least six months after symptoms. Furthermore, concerning the anti-NC IgG response, we found that in addition to those four individuals, three others also did not produce anti-NC IgG antibodies. All of them had a confirmed diagnosis of COVID-19 at least 6 months before inclusion in the study. We were not able to find a pattern for these diverse results. However, it is known that the magnitude of the humoral response seems to depend on the duration and magnitude of viral antigen exposure (26,27).

Additionally, although all tests for the detection of anti-SARS-CoV-2 antibodies were highly specific, we observed differences in test sensitivity concerning the detection of spike and nucleocapsid proteins. We emphasize that all serological tests used have sensitivity levels above 90%, as previously determined in tests with a significant number of samples. The differences observed may be due to the clinical profile of convalescent individuals, as well as the time elapsed after infection and the small sample number.

The cellular and humoral immunity components are both important to mediate immune protection. Additionally, it has been shown that recovered COVID-19 patients, even those with undetectable serum anti-SARS-CoV-2 IgG, are capable of presenting specific T cell immunity (28). In fact, we observed that all samples positive for the presence of specific antibodies were also positive for specific T cells, while samples negative for the presence of antibodies showed reactivity to at least one SARS-CoV-2 protein used as a stimulus. When interpreting this result, it should be considered that antibody investigation is performed by measuring peripheral blood without previous *in vitro* stimulation, while the measurement of cellular response requires *in vitro* stimulation, with reactivation of a previously sensitized system by contact with the antigen. This fact makes cellular response assessment a more sensitive method for measuring response specificity. Thus, these data suggest that even when antibody levels are reduced after a time, recovered individuals have the potential to produce an IFN-γ response after adequate antigenic stimulation.

Comparing sera from patients with severe COVID-19 and convalescent patients for anti-NC IgG and anti-S-RBD IgG by recombinant ELISA, Ni et al. (18) observed no significant differences in anti-NC and S-RBD IgG detection. The same group stimulated peripheral blood mononuclear cells (PBMCs) with recombinant nucleocapsid and S-RBD proteins by ELISpot, and there were no SARS-CoV-2-specific T cell IFN-γ responses in all severe patients tested, whereas one out of ten T cells on average could secrete IFN-γ after exposure to NC protein in convalescent individuals (18).

We also observed that most individuals in the control group did not present antibodies against SARS-CoV-2 antigens, while the production of IFN-γ could be detected by stimulation with at least one of the tested antigens, mainly by stimulating with recombinant proteins but not peptides. Pre-existing cross-reactive immunity has been reported in the literature and may be related to previous infection of individuals with seasonal coronaviruses, known to cause the common cold in humans, which could share partial sequence homology with SARS-CoV-2. It has been reported that between 20 and 50% of tested people, with no report of prior contact with SARS-CoV-2, present T cell reactivity to SARS-CoV-2 sequences (15,17,29,30). Since recombinant proteins contain portions exceedingly larger than specific previously selected regions of peptides, it is reasonable to assume a higher probability of finding portions common to other coronaviruses in protein samples, which could explain a greater number of negative controls capable of recognizing portions of these proteins. In addition, cut-off values of serological assays have been standardized according to the local population immune background.

**Figure S3:**
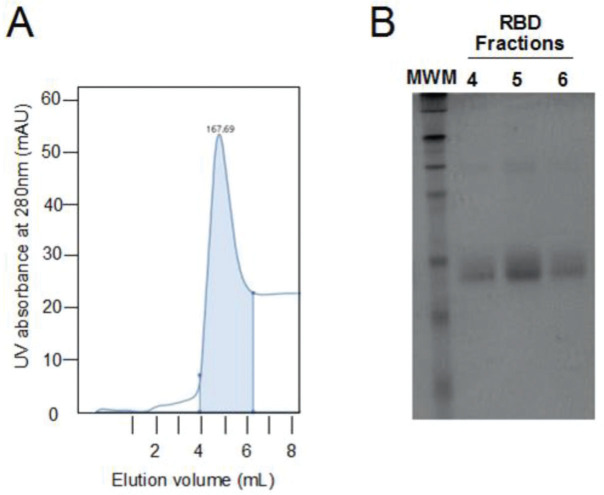
Chromatographic purification and SDS-PAGE of the SARS-CoV-2 RBD protein (Wuhan strain). The full-length SARS-CoV-2 RBD protein was purified through affinity chromatography (A). UV absorbance at 280 nm (mAU) was monitored over time, and fractions corresponding to the RBD protein (blue area in the graph) were collected. Protein purity was assessed through electrophoresis of the final collected fractions (B), showing that the RBD protein exhibits a molecular weight of 30.6 kDa. The molecular weight marker (MWM) used was the ColorPlus Prestained Protein Marker, Broad Range (7-175 kDa) (Thermo Fisher Scientific).

Another possibility is the presence of co-purified contaminant proteins and/or endotoxins in antigen samples that could interfere with assay results. In view of this possibility, we evaluated the presence of endotoxin in antigen samples. A qualitative test was positive for the presence of endotoxin in NC and RBD protein samples and negative for S1 protein and peptide samples (Table S1), although there are no contaminating proteins co-purified in the RBD protein (Figure S3). Considering that we did not observe differences in the response profile among the proteins tested, we can speculate that the presence of endotoxin does not appear to interfere with the IFN-γ response to protein stimuli.

In this context, even if we considered only the results of cellular response to stimulus with peptides and S1 protein (free of endotoxin), we observed that it is possible to detect a response to the pool of peptides and/or to S1 in almost all samples tested, including samples from uninfected controls, whose serology results were negative. In addition, we also detected the cellular response from convalescent samples whose serum collected at the time of inclusion in the study had a negative result for the tested antibodies.

We are aware that our sample size is a substantial limitation of this study, as well as the variability in volunteers’ age, elapsed time between infection and blood collection, and clinical form of convalescents, which varied among asymptomatic, mild symptoms, moderate and severe forms. While these are notable drawbacks, they were not a barrier to our findings.

Finally, the purpose of this study was to preliminarily understand the differences between cellular and humoral responses in COVID-19 within convalescent and unexposed individuals. We should note the possible genetic influence on SARS-CoV-2 susceptibility or protection, whereas the degree of exposure and immunity individuality may play a different role against infection.

## CONCLUSION

Taken together, the data obtained suggest that SARS-CoV-2 recombinant proteins seem to be specific for screening the humoral response of recovered individuals. On the other hand, these proteins appear to stimulate IFN-γ production by cells from convalescent individuals and from individuals with no history of contact with SARS-CoV-2. In contrast, stimulation with a pool of SARS-CoV-2 peptides is able to distinguish the cellular immune response of convalescents from that of unexposed individuals.

## AUTHOR CONTRIBUTIONS

Duarte AJS, Oshiro TM, Almeida A and Silva LT designed the research. Silva LT, Ortega MM, Tiyo BT, Oliveira LMS and Teixeira FME were responsible for the standardization of cellular assays. Silva LT, Ortega MM and Tiyo BT performed cellular assays. Viana IFT, Lima TE, Lins RD, Tozetto-Mendonza TR and Mendes-Correa MC performed serological tests. Silva LT, Ortega MM and Tiyo BT organized and analyzed the data. Oshiro TM, Silva LT, Ortega MM, Tiyo BT and Viana IFT wrote the manuscript. Oshiro TM and Duarte AJS coordinated and supervised the study. Duarte AJS and Oshiro TM were responsible for the funding acquisition. All authors read and approved the final version of the manuscript.

## Figures and Tables

**Table 1:** Demographic and clinical characteristics of individuals enrolled in this study.

Subject code	Age (years)^a^	Sex	SARS-CoV-2 RT-PCR	Anti-SARS-CoV-2 IgG at diagnosis	Time from COVID-19 diagnosis to study entry (months)^a^	Anti-SARS-CoV-2 IgG at study inclusion (UA/mL)^a^	Disease severity
CONV_1	22	Male	Positive	Positive	6	16.3	Mild symptoms
CONV_3	55	Male	Positive	Positive	10	95.3	Moderate symptoms with hospitalization
CONV_5	32	Female	Positive	Positive	7	26.9	Asymptomatic
CONV_6	75	Female	Not tested	Positive	9	63	Mild symptoms
CONV_7	29	Male	Positive	Positive	9	67.8	Moderate symptoms without hospitalization
CONV_9	72	Male	Positive	Positive	2	99.5	Mild symptoms
CONV_10	39	Male	Positive	Positive	2	44.7	Mild symptoms
CONV_11	42	Female	Not tested	Positive	6	Negative	Mild symptoms
CONV_12	30	Female	Positive	Positive	6	Negative	Mild symptoms
CONV_13	48	Female	Positive	Positive	1	Negative	Mild symptoms
CONV_14	41	Female	Positive	Positive	7	37.5	Mild symptoms
CONV_15	64	Female	Positive	Positive	7	438	Moderate symptoms with hospitalization
CONV_16	49	Female	Positive	Positive	6	22.3	Moderate symptoms without hospitalization
CONV_17	47	Female	Positive	Positive	1	Negative	Mild symptoms
**MEDIAN (IQ)**	**45 (33.7-53.5)**	**M (35.7%); F (64.3%)**	**-**	**-**	**6 (3-10)**	**54 (29.5-88.4)**	**-**
UI/UV_18	37	Male	Not tested	-	-	Negative	-
UI/UV_19	30	Female	Not tested	-	-	Negative	-
UI/UV_20	26	Female	Not tested	-	-	Negative	-
UI/UV_21	28	Female	Not tested	-	-	Negative	-
UI/UV_22	23	Female	Not tested	-	-	Negative	-
UI/UV_23	28	Female	Not tested	-	-	Negative	-
UI/UV_25	38	Female	Not tested	-	-	Negative	-
UI/UV_26	26	Female	Not tested	-	-	Negative	-
UI/UV_27	23	Female	Not tested	-	-	Negative	-
UI/UV_28	26	Male	Not tested	-	-	Negative	-
UI/UV_29	23	Female	Not tested	-	-	Negative	-
UI/UV_30	26	Male	Not tested	-	-	Negative	-
MEDIAN (IQ)	26** (25.2-28.5)	M (25%); F (75%)	-	-	-	-	-

a Median (25% and 75% IQ, interquartile values) for age, time from COVID-19 diagnosis to study entry and anti-SARS-CoV-2 IgG at study inclusion are shown. CONV, convalescent COVID-19 individual; UI/UV, uninfected/unvaccinated individual; M, male; F, female. Differences that were statistically significant were observed between the average ages of the studied groups,

** *p*<0.005. T-tests and nonparametric Mann-Whitney tests were used to calculate p values.

**Table 2:** Humoral and cellular responses of COVID-19 convalescent individuals and uninfected/unvaccinated donors to SARS-CoV-2 antigens.

Individual	Cell-mediated immunity				Humoral immunity		
	Peptide pool	S1	RBD	NC	S1/S2	RBD	NC
*Convalescent*							
1	+	NA	+	NA	+	+	+
3	+	+	+	+	+	+	+
5	NA	+	-	+	+	+	-
6	NA	NA	+	NA	+	+	+
7	+	-	+	+	+	+	+
9	+	NA	+	NA	+	+	+
10	NA	NA	-	NA	+	+	+
11	+	+	NA	-	-	-	-
12	+	+	NA	-	-	+	-
13	+	NA	+	NA	-	-	-
14	+	NA	+	NA	+	+	-
15	NA	+	NA	-	+	+	+
16	NA	+	NA	+	+	+	-
17	+	NA	+	NA	-	-	-
*Uninfected/unvaccinated*							
18	-	NA	+	NA	-	-	-
19	-	+	+	+	-	-	-
20	-	+	+	NA	-	-	-
21	+	NA	+	NA	-	-	-
22	+	NA	+	NA	-	-	-
23	-	NA	-	NA	-	-	-
25	-	+	+	+	-	-	-
26	-	NA	+	NA	-	-	-
27	-	+	+	NA	-	-	-
28	-	+	-	NA	-	-	-
29	-	NA	+	NA	-	+	-
30	+	NA	+	NA	-	-	-

The cellular immune response to a peptide pool or individual S1, RBD or NC proteins of SARS-CoV-2 was defined by the presence (+) or absence (-) of IFN-γ production by T cells. The humoral immune response was defined by the presence (+) or absence (-) of anti-SARS-CoV-2 S1/S2, RBD or NC IgG antibody titers. NA, not analyzed.

## APPENDIX

**Table S1:** Detection of endotoxin in SARS-CoV-2 antigens.

Sample	Result
Spike protein (S1)	-
Nucleocapsid protein (NC)	+
RBD protein	+
Membran peptide (M)	-
Nucleocapsid peptide (N)	-
Spike peptide (S)	-
Negative control	-
Positive control	+

Limulus Amebocyte Lysate (LAL) Gel Clot Reagent (LGC Biotecnologia Ltd.) was used to obtain the results regarding bacterial endotoxins.

## References

[1] Singhal T (2020). A Review of Coronavirus Disease-2019 (COVID-19). Indian J Pediatr.

[2] Li G, Fan Y, Lai Y, Han T, Li Z, Zhou P (2020). Coronavirus infections and immune responses. J Med Virol.

[3] Bacher P, Rosati E, Esser D, Martini GR, Saggau C, Schiminsky E (2020). Low-Avidity CD4+ T Cell Responses to SARS-CoV-2 in Unexposed Individuals and Humans with Severe COVID-19. Immunity.

[4] Kusnadi A, Ramírez-Suástegui C, Fajardo V, Chee SJ, Meckiff BJ, Simon H (2021). Severely ill COVID-19 patients display impaired exhaustion features in SARS-CoV-2-reactive CD8+ T cells. Sci Immunol.

[5] Rabaan AA, Al-Ahmed SH, Haque S, Sah R, Tiwari R, Malik YS (2020). SARS-CoV-2, SARS-CoV, and MERS-COV: A comparative overview. Infez Med.

[6] Rabaan AA, Al-Ahmed SH, Garout MA, Al-Qaaneh AM, Sule AA, Tirupathi R (2021). Diverse Immunological Factors Influencing Pathogenesis in Patients with COVID-19: A Review on Viral Dissemination, Immunotherapeutic Options to Counter Cytokine Storm and Inflammatory Responses. Pathogens.

[7] Yang L, Liu S, Liu J, Zhang Z, Wan X, Huang B (2020). COVID-19: immunopathogenesis and Immunotherapeutics. Signal Transduct Target Ther.

[8] Sekine T, Perez-Potti A, Rivera-Ballesteros O, Strålin K, Gorin JB, Olsson A (2020). Robust T Cell Immunity in Convalescent Individuals with Asymptomatic or Mild COVID-19. Cell.

[9] Griffin DE (2020). Are T cells helpful for COVID-19: the relationship between response and risk. J Clin Invest.

[10] Rosendahl Huber S, van Beek J, de Jonge J, Luytjes W, van Baarle D (2014). T cell responses to viral infections - opportunities for Peptide vaccination. Front Immunol.

[11] Zelba H, Worbs D, Harter J, Pieper N, Kyzirakos-Feger C, Kayser S (2021). A Highly Specific Assay for the Detection of SARS-CoV-2-Reactive CD4+ and CD8+ T Cells in COVID-19 Patients. J Immunol.

[12] Zhao J, Yuan Q, Wang H, Liu W, Liao X, Su Y (2020). Antibody Responses to SARS-CoV-2 in Patients With Novel Coronavirus Disease 2019. Clin Infect Dis.

[13] Bonifacius A, Tischer-Zimmermann S, Dragon AC, Gussarow D, Vogel A, Krettek U (2021). COVID-19 immune signatures reveal stable antiviral T cell function despite declining humoral responses. Immunity.

[14] Braun J, Loyal L, Frentsch M, Wendisch D, Georg P, Kurth F (2020). SARS-CoV-2-reactive T cells in healthy donors and patients with COVID-19. Nature.

[15] Grifoni A, Weiskopf D, Ramirez SI, Mateus J, Dan JM, Moderbacher CR (2020). Targets of T Cell Responses to SARS-CoV-2 Coronavirus in Humans with COVID-19 Disease and Unexposed Individuals. Cell.

[16] Law JC, Koh WH, Budylowski P, Lin J, Yue F, Abe KT (2021). Systematic Examination of Antigen-Specific Recall T Cell Responses to SARS-CoV-2 versus Influenza Virus Reveals a Distinct Inflammatory Profile. J Immunol.

[17] Le Bert N, Tan AT, Kunasegaran K, Tham CYL, Hafezi M, Chia A (2020). SARS-CoV-2-specific T cell immunity in cases of COVID-19 and SARS, and uninfected controls. Nature.

[18] Ni L, Cheng ML, Feng Y, Zhao H, Liu J, Ye F (2021). Impaired Cellular Immunity to SARS-CoV-2 in Severe COVID-19 Patients. Front Immunol.

[19] Thieme CJ, Anft M, Paniskaki K, Blazquez-Navarro A, Doevelaar A, Seibert FS (2020). Robust T Cell Response Toward Spike, Membrane, and Nucleocapsid SARS-CoV-2 Proteins Is Not Associated with Recovery in Critical COVID-19 Patients. Cell Rep Med.

[20] Thieme CJ, Anft M, Paniskaki K, Blazquez-Navarro A, Doevelaar A, Seibert FS (2020). The SARS-CoV-2 T-cell immunity is directed against the spike, membrane, and nucleocapsid protein and associated with COVID 19 severity. MedRxiv.

[21] Amanat F, Stadlbauer D, Strohmeier S, Nguyen THO, Chromikova V, McMahon M (2020). A serological assay to detect SARS-CoV-2 seroconversion in humans. Nat Med.

[22] Tozetto-Mendoza TR, Kanunfre KA, Vilas-Boas LS, Sanchez Espinoza EP, Paião HGO, Rocha MC (2021). Nucleoprotein-based ELISA for detection of SARS-COV-2 IgG antibodies: Could an old assay be suitable for serodiagnosis of the new coronavirus?. J Virol Methods.

[23] Guermonprez P, Valladeau J, Zitvogel L, Thery C, Amigorena S (2002). Antigen presentation and T cell stimulation by dendritic cells. Annu Rev Immunol.

[24] Zhan Y, Carrington EM, Zhang Y, Heinzel S, Lew AM (2017). Life and Death of Activated T Cells: How Are They Different from Naïve T Cells?. Front Immunol.

[25] Sela-Culang I, Kunik V, Ofran Y (2013). The structural basis of antibody-antigen recognition. Front Immunol.

[26] Long QX, Liu BZ, Deng HJ, Wu GC, Deng K, Chen YK (2020). Antibody responses to SARS-CoV-2 in patients with COVID-19. Nat Med.

[27] Seow J, Graham C, Merrick B, Acors S, Pickering S, Steel KJA (2020). Longitudinal observation and decline of neutralizing antibody responses in the three months following SARS-CoV-2 infection in humans. Nat Microbiol.

[28] Schwarzkopf S, Krawczyk A, Knop D, Klump H, Heinold A, Heinemann FM (2021). Cellular Immunity in COVID-19 Convalescents with PCR-Confirmed Infection but with Undetectable SARS-CoV-2-Specific IgG. Emerg Infect Dis.

[29] de Vries RD (2020). SARS-CoV-2-specific T-cells in unexposed humans: presence of cross-reactive memory cells does not equal protective immunity. Signal Transduct Target Ther.

[30] Mateus J, Grifoni A, Tarke A, Sidney J, Ramirez SI, Dan JM (2020). Selective and cross-reactive SARS-CoV-2 T cell epitopes in unexposed humans. Science.

